# Author Correction: Potential health and economic impacts of dexamethasone treatment for patients with COVID-19

**DOI:** 10.1038/s41467-021-22038-x

**Published:** 2021-03-05

**Authors:** Ricardo Águas, Adam Mahdi, Rima Shretta, Peter Horby, Martin Landray, Lisa White, the CoMo Consortium, Fatima Arifi, Chynar Zhumalieva, Inke N. D. Lubis, Antoninho Benjamin Monteiro, Ainura Moldokmatova, Siyu Chen, Aida Estebesova, Mofakhar Hussain, Dipti Lata, Emmanuel A. Bakare, Biniam Getachew, Mohammad Nadir Sahak, Phetsavanh Chanthavilay, Akindeh M. Nji, Yu Nandar Aung, Nathaniel Hupert, Sai Thein Than Tun, Wirichada Pan-Ngum, K. C. Sarin, Handoyo Harsono, Sana Eybpoosh, Renato Mendes Coutinho, Semeeh A. Omoleke, Amen-Patrick Nwosu, Nantasit Luangasanatip, Ainura Kutmanova, Aizhan Dooronbekova, Antonio Ximenes, Merita Monteiro, Olivier Celhay, Keyrellous Adib, Amel H. Salim, Yuki Yunanda, Mahnaz Hossain Fariba, Amirah Azzeri, Penny Hancock, Hakim Bekrizadeh, Sayed Ataullah Saeedzai, Ivana Alona, Grace Wezi Mzumara, Joao Martins, Jose L. Herrera-Diestra, Hamid Sharifi, Talant Abdyldaev, Babak Jamshidi, Noran Naqiah Hairi, Naima Nasir, Rashid U. Zaman, Sopuruchukwu Obiesie, Roberto A. Kraenkel, Nicholas Letchford, Lucsendar Raimunda Fernandes Alves, Sandra Adele, Lorena Suárez-Idueta, Nicole Advani, Manar Marzouk, Viviana Mabombo, Aibek Mukambetov, Adeniyi Kolade Aderoba, Bpriya Lakshmy Tbalasubramaniam, Nicole Feune de Colombi, Maria Angela Varela Niha, Francisco Obando, Parinda Wattanasri, Sompob Saralamba, Fatiha Shabaruddin, Shafiun Nahin Shimul, Maznah Dahlui, Reshania Naidoo, Caroline Franco, Michael G. Klein, Aisuluu Kubatova, Nusrat Jabin, Shwe Sin Kyaw, Luzia Tomas Freitas, Sunil Pokharel, Proochista Ariana, Chris Erwin G. Mercado, Shamil Ibragimov, John Robert C. Medina, Mesulame Namedre

**Affiliations:** 1 Mahidol-Oxford Tropical Medicine Research Unit, Nuffield Department of Medicine, University of Oxford, Oxford, UK; 2 Institute of Biomedical Engineering, Department of Engineering Science, University of Oxford, Oxford, UK; 3 Big Data Institute, Li Ka Shing Centre for Health Information and Discovery, Nuffield Department of Medicine, University of Oxford, Oxford, UK; 4 Centre for Tropical Medicine and Global Health, Nuffield Department of Medicine, University of Oxford, Oxford, UK; 5 Medical Research Council Population Health Research Unit at the University of Oxford, Nuffield Department of Population Health, Oxford, UK; 6 World Health Organization, Kabul, Afghanistan; 7 Public Fund “Institution of social development” in the Kyrgyz Republic, Bishkek, Kyrgyz Republic; 8 Department of Paediatrics, Faculty of Medicine, Universitas Sumatera Utara, Medan, Indonesia; 9 Universidade da Paz, Dili, Timor-Leste; 10 USAID Mission in the Kyrgyz Republic, Bishkek, Kyrgyz Republic; 11 Institute of Health Policy, Management and Evaluation, University of Toronto, Toronto, Canada; 12 Oxford Policy Management, Lalitpur, Nepal; 13 Department of Mathematics, Federal University, Oye-Ekiti, Ekiti State Nigeria; 14 Stop Transmission of Polio Program (STOP) program, World Health Organization, Birnin Kebbi, Nigeria; 15 Institute of Research and Education Development, University of Health Sciences, Vientiane, Lao PDR; 16 Laboratory for Public Health Research biotechnology, Biotechnology Center, University of Yaounde, Yaounde, Cameroon; 17 London School of Economics, London, UK; 18 Department of Medicine, Weill Cornell Medicine, and Cornell Institute for Disease and Disaster Preparedness, Cornell University, Ithaca, USA; 19 Mathematical And Economic MODelling Group, Mahidol-Oxford Tropical Medicine Research Unit, Faculty of Tropical Medicine, Mahidol University, Bangkok, Thailand; 20 Health Intervention and Technology Assessment Program (HITAP), Ministry of Public Health, Nonthaburi, Thailand; 21 COVID-19 Task Force for North Sumatera province, Medan, Indonesia; 22 Department of Epidemiology and Biostatistics, Research Centre for Emerging and Reemerging Infectious Diseases, Pasteur Institute of Iran, Tehran, Iran; 23 Center for Mathematics, Computation and Cognition, Federal University of ABC, São Paulo, Brazil; 24 World Health Organization, Birnin Kebbi, Nigeria; 25 Nuffield Department of Medicine, University of Oxford, Oxford, UK; 26 Ministry of Public Health of the Kyrgyz Republic, Bishkek, Kyrgyz Republic; 27 Universidade Nacional Timor Lorosae, Dili, Timor-Leste; 28 Menzies School of Health Research, Charles Darwin University, Darwin, Australia; 29 Independent researcher (no affiliation), Ithaca, NY USA; 30 University of Nottingham, Nottingham, UK; 31 Population Services International, Addis Ababa, Ethiopia; 32 Department of Community Medicine, Faculty of Medicine, Universitas Sumatera Utara, Medan, Indonesia; 33 Government of People’s Republic of Bangladesh, Dhaka, Bangladesh; 34 Department of Research, Development and Innovation, University of Malaya Medical Centre, Kuala Lumpur, Malaysia; 35 Department of Primary Care, Faculty of Medicine and Health Sciences, Universiti Sains Islam Malaysia (USIM), Kuala Lumpur, Malaysia; 36 Department of Statistics, Payame Noor University, Tehran, Iran; 37 Ministry of Public Health, Kabul, Afghanistan; 38 Malawi Ministry of Health, Lilongwe, Malawi; 39 Centro de Simulacion y Modelos (CeSiMo), Universidad de Los Andes, Merida, Venezuela; 40 HIV/STI Surveillance Research Center, and WHO Collaborating Center for HIV Surveillance, Institute for Futures Studies in Health, Kerman University of Medical Sciences, Kerman, Iran; 41 Medical Association in the Kyrgyz Republic, Bishkek, Kyrgyz Republic; 42 Department of Biostatistics, Kermanshah University of Medical Sciences, Kermanshah, Iran; 43 University of Malaya, Kuala Lumpur, Malaysia; 44 Oxford Policy Management, Oxford, UK; 45 Centre for Tropical Medicine and Global Health, Nuffield Department of Medicine, University of Oxford, Oxford, UK; 46 Institute for Theoretical Physics, São Paulo State University (UNESP), São Paulo, Brazil; 47 Oxford Policy Management, Oxford, UK; 48 University College of London, London, UK; 49 Valid International Ltd, Oxford, UK; 50 Soros Foundation in the Kyrgyz Republic, Bishkek, Kyrgyz Republic; 51 Ministry of Health Timor-Leste, Dili, Timor-Leste; 52 PEAK Urban and Oxford Martin Informal Cities Programmes, COMPAS, School of Anthropology and Museum Ethnography, University of Oxford, Oxford, UK; 53 Faculty of Pharmacy, University of Malaysia, Kuala Lumpur, Malaysia; 54 Institute of Health Economics, University of Dhaka, Dhaka, Bangladesh; 55 Department of Social and Preventive Medicine, Faculty of Medicine, University of Malaya, Kuala Lumpur, Malaysia; 56 Department of Marketing & Business Analytics, Lucas College and Graduate School of Business, San José State University, San José, USA; 57 Independent researcher (no affiliation), Manila, Philippines; 58 Department of Epidemiology and Biostatistics, College of Public Health, University of the Philippines Manila, Manila, Philippines; 59 Fiji Center for Disease Control (CDC), Suva, Fiji

**Keywords:** SARS-CoV-2, Viral infection, Health care economics, Epidemiology

Correction to: *Nature Communications* 10.1038/s41467-021-21134-2, published online 10 February 2021.

The original version of this Article contained an error in the author affiliations.

The affiliation of Amirah Azzeri with Department of Primary Care, Faculty of Medicine and Health Sciences, Universiti Sains Islam Malaysia (USIM) was inadvertently omitted.

This has now been corrected in both the PDF and HTML versions of the Article.

