# Supplementary material for: Potential health and economic impacts of dexamethasone treatment for patients with COVID-19
Source: Nat Commun. 2021 Feb 10;12:915. doi: 10.1038/s41467-021-21134-2 (PMC7875992; doi:10.1038/s41467-021-21134-2)
Supplement: Supplementary file 1 — Supplementary Information [file 41467_2021_21134_MOESM1_ESM.docx]

**Potential health and economic impacts of dexamethasone treatment for patients with COVID-19 – Supplementary Information**

Ricardo Águas, Adam Mahdi, Rima Shretta, Peter Horby, Martin Landray, Lisa White,

and the CoMo Consortium

**Supplementary Table 1.** Age structured values for the UK population, *n*^1^, the infection hospitalisation rate, $p_{H}$^2^, and the normalised hospitalisation fatality rate, $p_{F}$^2^.

| Age group (years) | Population  (*n*) | $\boldsymbol{p}_{\boldsymbol{H}}$ | $\boldsymbol{p}_{\boldsymbol{F}}$ |
| --- | --- | --- | --- |
| 0-4 | 3,924,490 | 0.002 | 0.019 |
| 5-9 | 4,119,566 | 0.002 | 0.019 |
| 10-14 | 3,956,340 | 0.002 | 0.019 |
| 15-19 | 3,686,133 | 0.002 | 0.019 |
| 20-24 | 4,074,640 | 0.006 | 0.035 |
| 25-29 | 4,484,067 | 0.006 | 0.035 |
| 30-34 | 4,706,828 | 0.013 | 0.060 |
| 35-39 | 4,588,196 | 0.013 | 0.060 |
| 40-44 | 4,308,130 | 0.017 | 0.10 |
| 45-49 | 4,296,121 | 0.017 | 0.10 |
| 50-54 | 4,634,540 | 0.035 | 0.21 |
| 55-59 | 4,538,925 | 0.035 | 0.21 |
| 60-64 | 3,905,016 | 0.071 | 0.40 |
| 65-69 | 3,381,761 | 0.071 | 0.40 |
| 70-74 | 3,388,488 | 0.113 | 0.66 |
| 75-79 | 2,442,147 | 0.113 | 0.66 |
| 80-84 | 1,736,567 | 0.32 | 1 |
| 85-89 | 1,077,555 | 0.32 | 1 |
| 90-94 | 490,577 | 0.32 | 1 |
| 95-99 | 130,083 | 0.32 | 1 |
| 100+ | 15,834 | 0.32 | 1 |

**Supplementary Table 2:** Cost inputs and assumptions used.

| Variable | Value (GBP) |
| --- | --- |
| Dexamethasone treatment course | 5.00 |
| Cost per day for patients on mechanical ventilation in an ICU | 4,520.00 |
| Cost per day for patients on non-invasive ventilation with oxygen | 1,356.00 |
| Incremental number of days spent in hospital by survivors | 4.5 |

**Supplementary Table 3:** Sensitivity of the main epidemiological and costing metrics to assumptions on the infection hospitalisation rate, $p_{H}$, and the normalised hospitalisation fatality rate, $p_{F}$. The two countries from which p_h_ and p_f_ vectors are derived and corresponding median and 90% confidence intervals are shown in the columns.

|  | France | Spain |
| --- | --- | --- |
| Lives Saved | 12,000  [4,250 – 27,000] | 5,200  [2,000 – 12,000] |
| Life Years Gained | 102,000  [37,000 – 236,000] | 45,000  [16,500 – 105,000] |
| Total Incremental Cost (£) | 84,500,000  [5,700,000 – 330,000,000] | 37,500,000  [2,500,000 – 143,300,000] |
| Incremental Cost per Life Saved (£) | 8,230  [565 – 17, 700] | 8,200  [650 – 17,500] |
| Incremental Cost per Life Year Gained (£) | 940  [65 – 2,020] | 900  [63 – 1,950] |

**Supplementary Table 4.** Infection hospitalisation rate, $p_{H}S$, and the normalised hospitalisation fatality rate, $p_{F}S$, derived from Spanish data^3,4^.

| Age group (years) | $\boldsymbol{p}_{\boldsymbol{H}}\boldsymbol{S}$ | $\boldsymbol{p}_{\boldsymbol{F}}\boldsymbol{S}$ |
| --- | --- | --- |
| 0-4 | 0 | 0 |
| 5-9 | 0 | 0 |
| 10-14 | 0.001 | 0 |
| 15-19 | 0.001 | 0 |
| 20-24 | 0.005 | 0.038 |
| 25-29 | 0.005 | 0.038 |
| 30-34 | 0.012 | 0.041 |
| 35-39 | 0.012 | 0.041 |
| 40-44 | 0.016 | 0.055 |
| 45-49 | 0.016 | 0.055 |
| 50-54 | 0.030 | 0.103 |
| 55-59 | 0.030 | 0.103 |
| 60-64 | 0.048 | 0.234 |
| 65-69 | 0.048 | 0.234 |
| 70-74 | 0.078 | 0.533 |
| 75-79 | 0.078 | 0.533 |
| 80-84 | 0.121 | 1 |
| 85-89 | 0.121 | 1 |
| 90-94 | 0.121 | 1 |
| 95-99 | 0.121 | 1 |
| 100+ | 0.121 | 1 |

**Supplementary Information – references**

1 World Population Prospects 2019, <https://population.un.org/wpp/Download/Standard/Population/> (2020).

2 Salje, H. *et al.* Estimating the burden of SARS-CoV-2 in France. *Science*, eabc3517, doi:10.1126/science.abc3517 (2020).

3 Pollan *et al*. Prevalence of SARS-CoV-2 in Spain (ENE-COVID): a nationwide, population-based seroepidemiological study. *Lancet* **396**, 535-544, doi: 10.1016/S0140-6736(20)31483-5 (2020).

4 <https://www.mscbs.gob.es/profesionales/saludPublica/ccayes/alertasActual/nCov-China/documentos/Actualizacion_109_COVID-19.pdf> (2020).
